# Assessment of Knowledge and Attitude Toward Pelvic Inflammatory Disease Among Women in Saudi Arabia

**DOI:** 10.7759/cureus.45013

**Published:** 2023-09-11

**Authors:** Azharuddin Sajid Syed Khaja, Mubashir Zafar, Abdulrahman Musaad A Alshammari, Saqer Alharbi, Abdulkarim Muflih S Alghaithi, Badr Alshahri, Mohd Saleem, Nuzhat Parveen, Ghorashy Mohammed

**Affiliations:** 1 Department of Pathology, University of Hail College of Medicine, Hail, SAU; 2 Department of Family and Community Medicine, University of Hail College of Medicine, Hail, SAU; 3 Department of Obstetrics and Gynecology, University of Hail College of Medicine, Hail, SAU

**Keywords:** preventive practices, attitude, knowledge, sexually transmitted infections, pelvic inflammatory disease

## Abstract

Purpose

Pelvic inflammatory disease (PID) is a serious infection of the female reproductive system that can lead to long-term complications such as infertility, chronic pelvic pain, and ectopic pregnancy. PID is also associated with an increased risk of HIV infection and other sexually transmitted infections (STIs). Early diagnosis and treatment of PID is crucial to prevent complications. Despite the severe consequences of PID, many women are unaware of the risks associated with this condition. This lack of awareness can lead to delayed diagnosis and treatment, increasing the risk of complications. This study explores women's knowledge and attitudes regarding PID.

Methods

A cross-sectional survey was conducted using a bilingual, community-based questionnaire, circulated using different social media platforms. A total of 239 participants were selected through convenient non-probability sampling from the public in the Kingdom of Saudi Arabia. The collected data was analyzed using SPSS Statistics version 26 (IBM Corp. Released 2019. IBM SPSS Statistics for Windows, Version 26.0. Armonk, NY: IBM Corp.). The chi-square test was applied to determine the differences between knowledge and attitude levels with participants' socio-demographic characteristics. A p-value <0.005 was considered statistically significant.

Results

Appropriate PID knowledge level was found only in 32% of the respondents and was significantly associated with the respondents' family history of the PID (p=0.025). A positive attitude toward PID/STI was also observed only in 36% of the study participants, which was significantly associated with the respondents' age (p˂0.001), marital status (p˂0.001), occupation (p˂ 0.001), past medical/surgical history (p=0.006), and family history of the PID (p˂0.009).

Conclusion

The present study reported average levels of appropriate knowledge and attitudes toward PID among female respondents, which could be further improved by increasing PID/STI awareness programs.

## Introduction

Pelvic inflammatory disease (PID) can be defined as a clinical syndrome characterized by upper genital tract infection in women. Worldwide, a significant proportion of cases are caused by bacterial infections, such as *Chlamydia trachomatis* and *Neisseria gonorrhoeae* [1]. Several studies have also shown that the microorganisms found in the vaginal flora, such as gram-positive and gram-negative anaerobic organisms and aerobic/facultative gram-positive and gram-negative rods and cocci, have also been implicated in the pathogenesis of PID [2-4]. Women who have multiple sexual partners and are under the age of 25 and have a history of sexually transmitted infections (STIs) or use intrauterine devices are at a higher risk of developing PID [5-6]. The signs and symptoms of PID range from unnoticeable or subtle and mild to severe, including but not limited to having lower abdominal pain, mild pelvic pain, increased vaginal discharge, pelvic organ tenderness, irregular menstrual bleeding, fever (>38° C), inflammation, abdominal tenderness, cervical motion tenderness, painful and frequent or difficult urination, uterine tenderness, or adnexal tenderness [7]. When the symptoms are mild, PID can go unnoticed by women and their healthcare providers [8]. However, if diagnosed lately and left untreated, it can lead to many complications, such as ectopic pregnancy, tubal factor infertility, and chronic abdominal pain, and has been associated with ovarian cancer. Hence, it is one of the main causes of concern for reproductive diseases in women [9-10].

PID can be largely preventable through knowledge and adopting appropriate preventive practices among women. However, several studies have shown that knowledge, attitude, and preventive practices among women about PID are still inadequate, which can lead to delayed diagnosis and treatment. A study conducted at the Maternal and Child Minia University Hospital in Egypt among 100 women showed that 85% of women had poor knowledge regarding PID and its prevention at the pre-educational test. Furthermore, 83% of women had unacceptable reported practices related to post-abortion care at the pre-educational test [11]. Another study from the College of Applied Medical Science in Hafar Al-Batin, Kingdom of Saudi Arabia (KSA), assessed the level of knowledge on PID among adolescent girls and found that 54% of the girls had average knowledge, and 30% had good knowledge. In contrast, the lowest percentage (16%) of girls showed poor knowledge [12].

Raising awareness and knowledge among females about PID and its symptoms is crucial, as early detection can significantly reduce the chances of severe complications. The present study was designed to investigate the knowledge and attitude levels of Saudi women about PID.

## Materials and methods

Ethical approval

The survey was conducted after the ethical approval by the Medical Ethics Committee of the University of Hail (ethical approval code. H-2022-1055-19136).

Research survey design and target population

The present research is a cross-sectional analytical study and is conducted between December 2022 and May 2023 in KSA. The general female population of the KSA, female medical and dental students, and some patients from the University of Hail clinic were included as study participants. Females who are younger than 18 years were excluded from the study. The participants were selected through a convenient sampling technique. To determine the appropriate survey sample size, WHO recommendations for the minimal sample size needed for a prevalence study were utilized (https://apps.who.int/iris/handle/10665/40062) [13]. Parameters for sample size calculation were a margin of error of 5%, 95% confidence of interval, and 19% knowledge prevalence from the previous study; the required sample size was 229.

Data collection

Data was collected using an online self-administered questionnaire. The structured questionnaire consisted of respondents' demographic info and focused on questions testing their knowledge of PID, the side effects of contracting the disease, and the possible treatments, if any. The questionnaire was designed using Google Forms (Google LLC, Mountain View, California, United States), and its link was shared through social media apps. The questionnaire was prepared in English and Arabic languages. Participation in the survey was taken as the consent of the participants.

Study questionnaire

The e-survey questionnaire is divided into three sections: socio-demographic information of the respondents, in which they provide us information about their age, gender, occupation, educational level, etc. The second section contained 12 questions and focused on testing the respondent's knowledge regarding PID, like sources of information, mode of infection, symptoms, risks, presence of a cure or a vaccine, etc. Each question was answered "yes," "no," or "I don't know." Correct answers scored 1, and incorrect answers scored 0. The third section was composed of eight questions and measured the attitude toward STD/PID, like the right to seek professional help, social outcasting, presence of a possible group at risk, etc. Responses were recorded on a Likert scale (1, strongly agree; 2, agree; 3, undecided; 4, disagree; 5, strongly disagree). Total scores were calculated, the minimum score was 0, the maximum score was 12 for the knowledge scale, and the maximum score was 8 for the attitude scale. Participants who secured 50 percent correct responses were labeled as having appropriate knowledge and a positive attitude.

Statistical analysis

Data were entered in SPSS Statistics version 26.0 (IBM Corp. Released 2019. IBM SPSS Statistics for Windows, Version 26.0. Armonk, NY: IBM Corp). Descriptive analysis was applied to calculate the mean and standard deviation. Inferential statistics were used to calculate the difference between knowledge and attitude by socio-demographic characteristics. The chi-square test was used to find out significant differences between the groups. A p-value <0.05 was considered statistically significant.

## Results

Baseline characteristics of the study participants

In the present study, we collected responses from 239 female participants. The baseline characteristics of the study participants are presented in Table 1.

**Table 1:** Baseline characteristics of the study participants (n=239) PID: pelvic inflammatory disease

No	Characteristics	Frequency (n)	Proportion (%)
1	Age (mean ±SD) (years)	32 (±0.46)	
	18-30	161	67.4
	31-50	78	32.6
2	Marital status		
	Married	85	35.6
	Single	154	64.4
3	Education level		
	Primary	45	18.8
	Higher education	194	81.2
4	Occupation		
	Employed	72	30.2
	Student	132	55.2
	Housewife	35	14.6
5	Household income (SR)		
	Less than 3000	26	10.9
	3000-15000	138	57.7
	More than 15000	75	31.4
6	Past medical/surgical history		
	Yes	175	76.2
	No	64	23.8
7	Family history of PID		
	Yes	11	4.6
	No	228	95.4

The mean age of the participants was 32 (±0.46) years. About two-thirds (65%) of the participants were single and below the age of 30 years. A majority of the participants (80%) had higher education. More than half (55%) of the participants were students, and more than 75% of the participants had a past medical or surgical history of PID. Only 4.6% of participants had a family history of PID.

Knowledge of the participants toward PID

We designed 12 questions that assess the participants' knowledge of PID, and the results are shown in Table 2.

**Table 2:** Knowledge of PID among the study participants (n=239) PID: pelvic inflammatory disease, STI: sexually transmitted infection

Knowledge questions (correctly answered)	N (%)
Cause of PID	
A sexual relationship with an infected (STI) person	191 (78.9)
Mode of transmission of PID	
Multiple sexual partners	147 (61.5)
Long-term risk of PID	
Infertility	226 (70.65)
Short-term risk of PID	
Pain and ectopic pregnancy	119 (49.7)
A reliable source of PID	
Doctor	163 (68.2)
Social media	43 (18)
Family and friends	21 (8.8)
PID cure completely	
Yes	80 (33.4)
Attend the awareness program regarding PID	
Yes	20 (8.4)
A vaccine to prevent PID	
Yes	51 (21.3)
Avoid being in a sexual relationship with PID	
Yes	203 (84.9)
Adolescents are more vulnerable to PID	
Yes	61 (25.5)
Easy to identify STI patients in the community	
Yes	21 (8.8)
Should seek medical help for PID	
Yes	173 (72.4)

**Figure 1:**
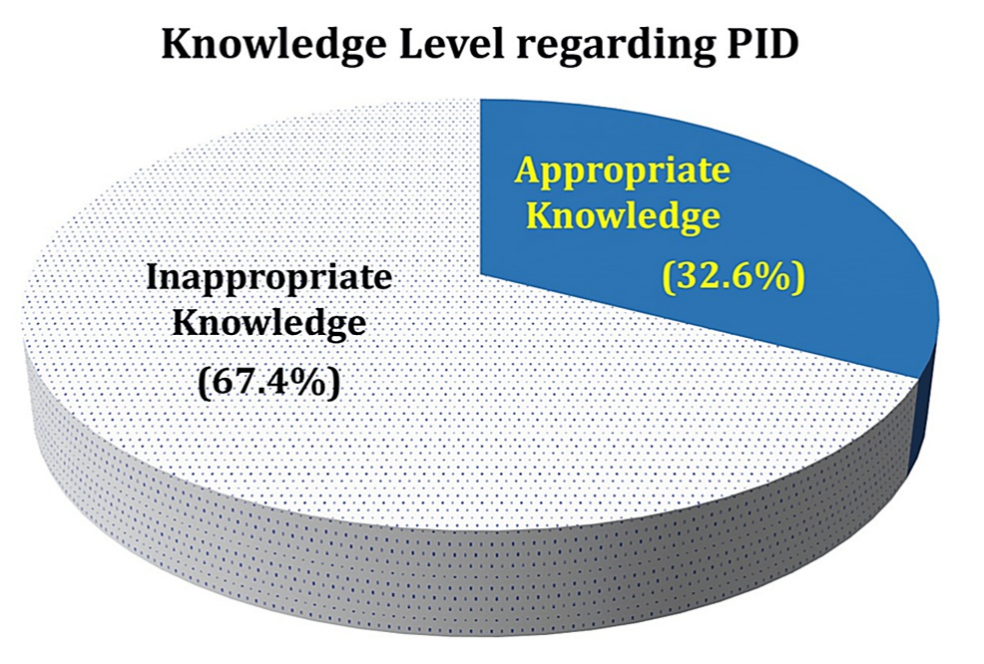
Knowledge level regarding PID among the study participants PID: pelvic inflammatory disease

In our survey, more than 67% of the participants had inappropriate knowledge of PID, while only 32% had appropriate knowledge regarding PID (Figure 1).

When asked about the possible cause of PID, almost 80% chose the "sexual relationship with infected person" option. More than 60% chose "multiple sexual partners" when questioned about the mode of transmission of PID. Almost 70% answered "infertility" as the long-term risk of PID, and nearly 50% picked pain and ectopic pregnancy as the short-term risk of PID. Almost 68% of participants believed doctors are a reliable source of information regarding PID. More than 84% agreed that avoiding being involved in a sexual relationship with an infected person is the best preventative measure. More than 72% agreed that an infected person should seek professional help.

Almost a third of the participants said yes when questioned about a cure for PID (33.4%), while two-thirds of the respondents did not believe that PID could be completely cured. However, very few participants (8.4%) attended any awareness programs regarding PID. Only around one-fifth of the female participants (21.3%) thought vaccination could prevent PID. On the other hand, most of the female participants (78.7%) were of the view that PID could not be prevented by vaccination, and a quarter of our sample agreed that adolescents are more vulnerable to PID/STI (25.5%). Less than 9% of the participants denied any difficulty in identifying infected persons in the community.

Attitude of the participants toward PID

**Figure 2:**
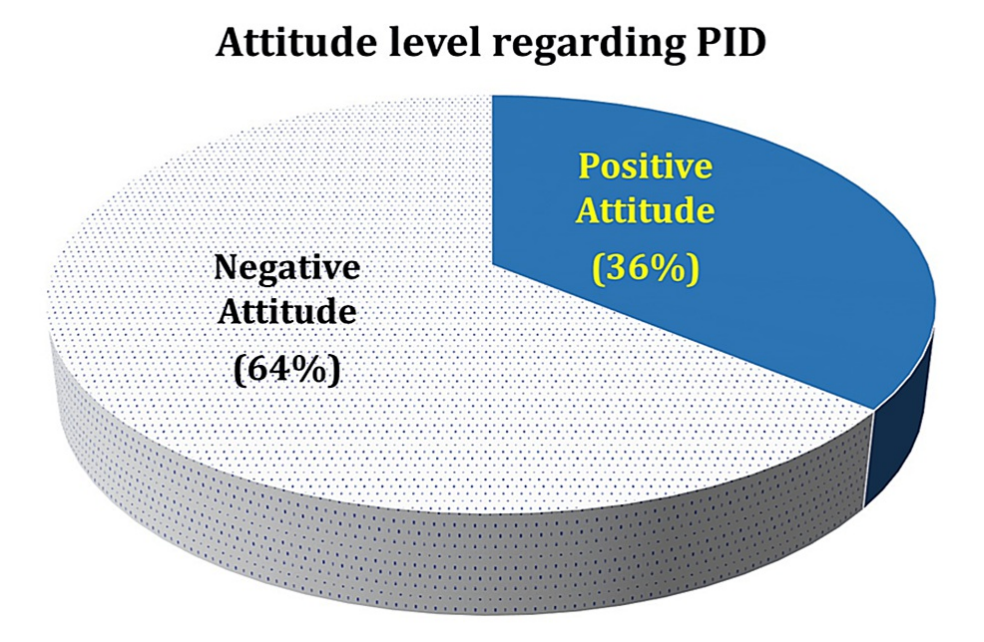
Attitude level regarding PID among the study participants PID: pelvic inflammatory disease

Attitude toward PID is another crucial factor influencing women's health-seeking behavior and their ability to prevent and manage this condition. In our survey, we questioned participants on their stance toward PID (Table 3) and found that only 36% of the participants had a positive attitude toward PID/STI (Figure 2).

**Table 3:** Attitude toward PID/STI among the study participants (n=239) PID: pelvic inflammatory disease, STI: sexually transmitted infection

Attitude questions (correctly answered)	N (%)
PID/STI caused social stigma in society (disagree)	110 (46)
Preventive measures for PID/STI (agree)	31 (13)
Seek treatment of PID/STI (agree)	113 (47.3)
Condom use during sexual relationship (agree)	40 (16.7)
Disclose the status of PID/STI with sexual partner (agree)	152 (63.6)
Safe sexual practice to prevent PID/STI (agree)	151 (63.1)
Screening for PID/STI is essential to all individuals (agree)	133 (55.6)
Need for awareness program regarding PID/STI (agree)	152 (63.6)

The data also showed that almost half of the participants (46%) disagreed that PID/STI causes a social stigma. While more than 47% agreed on seeking treatment for PID/STI, only 13% agreed on taking preventative measures for PID/STI. More than 16% believed in using a condom during sex. When asked about the disclosure of the status of PID/STI with the sexual partner, nearly 64% affirmed the disclosure. About 64% acknowledged practicing safe sex contributes to preventing PID/STI. More than half the sample (55.6%) accepted the need for screening tests for all individuals for PID/STI. Nearly 64% urged the need for more PID/STI awareness programs.

Relationship between knowledge level with socio-demographic characteristics of the study participants

Next, we calculated any correlation between knowledge level and socio-demographic features, as shown in Table 4.

**Table 4:** Relationship between knowledge level and socio-demographic characteristics among the participants (n=239) PID: pelvic inflammatory disease, STI: sexually transmitted infection

Characteristics	Good knowledge	Poor knowledge	p-value
Age (in years)			
18-30	50	111	0.3
31-50	28	50	
Marital status			
Married	31	54	0.348
Single	47	107	
Education level			
Primary	18	27	0.242
Higher education	60	134	
Occupation			
Employed	24	48	0.981
Student	43	89	
Housewife	11	24	
Household income (SR)			
Less than 3000	8	18	0.403
3000-15000	41	97	
More than 15000	29	46	
Past medical/surgical history			
Yes	25	139	0.2
No	53	122	
Family history of PID/STI			
Yes	7	4	0.025
No	71	157	

There were little to no significant associations of knowledge levels with the respondents' age, marital status, educational level, occupation, household income, and past medical/surgical history. However, we found a significant association between the family history of PID and PID/STI knowledge among the study participants, as participants with a positive family history of PID/STI have good knowledge compared to those with no family history of PID/STI (p=0.025).

Relationship between attitude level and socio-demographic characteristics of the study participants

In our study, we also analyzed differences between attitude levels and socio-demographic characteristics of the study participants (Table 5).

**Table 5:** Relationship between attitude level and socio-demographic characteristics among the participants (n=239) PID: pelvic inflammatory disease

Characteristics	Positive attitude	Negative attitude	p-value
Age			
18-30	46	115	˂0.001
31-50	40	38	
Marital status			
Married	43	42	˂0.001
Single	43	111	
Education level			
Primary	19	26	0.333
Higher education	67	127	
Occupation			
Employed	38	34	˂0.001
Student	28	104	
Housewife	20	15	
Household income (SR)			
Less than 3000	10	16	0.282
3000-15000	44	94	
More than 15000	32	43	
Past medical/surgical PID history			
Yes	32	32	0.006
No	54	121	
Family history of PID			
Yes	8	3	0.009
No	78	150	

There were no significant correlations between attitude levels of PID/STI and the respondents' educational status (p=0.333) or household income levels (p=0.282). However, participants below 30 years of age had a negative attitude compared to participants above 30, and this difference was statistically significant (p˂0.000). There was also considerable evidence that marital status could also affect the attitude toward PID/STI, as married participants had a positive attitude toward PID/STI compared to participants who were single or unmarried (p˂0.000).

In our survey, students had a negative attitude toward PID/STI compared to employed and housewife participants (p=0.000). We also noticed that participants with a past medical/surgical PID history had a positive attitude toward PID/STI compared to those who did not have a past medical/surgical PID history (p=0.006). Moreover, there was a statistically significant association between a family history of PID/STI and attitude levels toward PID/STI, as respondents with a family history of PID had a positive attitude toward PID/STI compared to those respondents with no PID family history (p=0.009).

## Discussion

PID is a severe infection that affects the female reproductive system and can result in long-term complications, including chronic pelvic pain, infertility, and ectopic pregnancy. In fact, PID is a leading cause of infertility in women, with up to 14% of women with PID experiencing infertility due to the infection [14]. Although PID is not related to elevated mortality, it is related to high morbidity. PID accounts for 94% of the morbidity associated with STIs in women (including HIV), as estimated by the WHO in established market economies. The interpretation is difficult due to the lack of validation studies and clarification of how the estimates were derived [15]. A significant problem of PID is thought to exist in women of reproductive age, but little is known about PID epidemiology in Gulf countries and other growing countries. The disease burden and associated risk factors are poorly understood but must be inspected to update public health action and clinical practice. Knowledge, attitude, and preventive practices among women are crucial in reducing the incidence of PID. However, the data on PID/STI in KSA and other Islamic countries are very limited. The current study assessed the knowledge, attitude, and awareness of STIs among women in KSA.

The results from our study demonstrated that only 32% of the females in the Hail region had appropriate knowledge of PID/STI, which is similar to the analysis performed in the Hafar Al-Batin region of KSA, where the authors reported that 30% of the women had good PID knowledge [12]. However, another study from Nigeria reported that 52% of the study participants had considerable knowledge of PID [16], which is more than the knowledge level of our study participants. This discrepancy can be attributed to the study population, as Akoko et al. conducted their study on female students in the College of Health Sciences. It is expected that the participants from the health science college would have some knowledge regarding PID compared to the general population. In contrast, our study population included not only medical and nursing students but also the general women population of the Hail region. Another study from Egypt reported that 85% of the female participants had poor knowledge regarding PID and its prevention in the pre-educational test. However, the post-educational test showed improvement, with 90% displaying good knowledge and prevention about PID [11].

In the current study, there were no significant associations of PID knowledge level with socio-demographic characteristics (age and marital status), similar to another cross-sectional survey from the Albaha region of KSA [17]. However, another study from Malaysia reported a significant correlation between PID knowledge and the age of the participants [18], which can be due to the grouping in the age category. Our study grouped the participants as "less than 30" and "more than 30" based on the age category, whereas all the participants were below the age of 30 in Folasayo et al.'s study. In our survey, we found a significant correlation between the family history of the participants and PID knowledge which can be due to the awareness of the disease in the family members of the infected individuals. However, steps should be taken to change this as awareness for STI/PID does not concern the sick only; rather, it concerns all of society. However, a big obstacle in raising awareness is that the topic of PID/STI is still taboo among the general population, and this can make it difficult to talk about STIs and how to prevent them. Moreover, there is a lack of access to education about PID/STI, and there are not enough sex education programs in schools, meaning young people may not learn about PID/STI until they are sexually active.

Attitude toward PID/STI is another important factor influencing women's health-seeking behavior and their ability to prevent and manage this condition. Negative attitudes toward discussing sexual health and seeking medical help can hinder effective prevention and early detection of PID. Studies have shown that cultural and societal barriers often prevent open discussions about sexual health, leading to a lack of awareness and delayed treatment-seeking behaviors [17-18]. Addressing these attitudes requires comprehensive sexual education programs and destigmatizing conversations about sexual health. In our study, positive attitudes regarding PID were observed among participants with increasing age, who are married, who had past medical/surgical PID/STDs, and participants with a positive family history of PID/STI. Studies have shown that cultural and societal barriers often prevent open discussions about sexual health, leading to a lack of awareness and delayed treatment-seeking behaviors. Addressing these attitudes requires comprehensive sexual education programs and destigmatizing conversations about sexual health. Women's attitudes toward PID/STI were also influenced by their social and cultural context. In some countries, women are stigmatized for having PID. Furthermore, there are very few studies on the attitude toward PID; thus, focusing on this would increase awareness and, ultimately, the attitude toward PID/STI.

Strength of the present study and key implications

The present study examines the knowledge and attitude levels of women regarding PID, which is considered to be taboo in a conservative society such as KSA. In fact, there are not many knowledge, attitude, and practice (KAP) studies on PID in the Middle East region. This study is valuable for identifying gaps in knowledge and areas where education and intervention regarding PID are needed, which can guide public health initiatives. Comprehensive sexual education is pivotal in equipping women with accurate information about PID and promoting preventive behaviors. School-based programs, community outreach initiatives, and online resources can provide education on safe sexual practices, STI prevention, and the importance of regular screening. Encouraging open dialogue about sexual health, STI testing, and condom use can help reduce the risk of PID transmission. PID is a preventable condition that requires increased knowledge, improved attitudes, and appropriate preventive practices among women. Promoting comprehensive sexual education, addressing barriers to healthcare access, and encouraging open communication between sexual partners are essential steps toward reducing the burden of PID.

Limitations of the study

Our study has some limitations. Firstly, self-reported data may be subject to recall and social desirability biases, leading to inaccurate information. Secondly, KAP studies often rely on small sample sizes and may not be representative of the entire population. Moreover, KAP studies provide only a snapshot of knowledge, attitudes, and practices at a specific time, limiting their ability to capture long-term changes. While KAP studies can provide valuable insights into women's knowledge and attitudes toward PID, they should be interpreted cautiously and complemented with other research methods. We also encountered difficulty discussing the topic of STIs/PID, as the study topic is considered taboo in a conservative society. Despite these limitations, KAP studies remain valuable in providing insights into PID-related factors but should be complemented with other research methods for a comprehensive understanding.

## Conclusions

In conclusion, PID is a serious health problem that can significantly affect women's reproductive health. The present study reported average levels of appropriate knowledge and attitude toward PID among the female respondents, which could be further improved by increasing PID/STI awareness programs. Knowledge, attitude, and preventive practices are important factors that influence women's health-seeking behavior and their ability to prevent and manage PID.
